# Difficult Conversation Case: Death Notification

**DOI:** 10.21980/J8.52354

**Published:** 2025-12-31

**Authors:** Charles Lei, Tiffany Moadel, Suzanne Bentley, Amrita Vempati, David Fernandez, Daniela Ortiz, Anita Rohra, Stephanie Stapleton, Hillary Moss

**Affiliations:** 1 Hennepin County Medical Center, Department of Emergency Medicine, Minneapolis, MN; 2 Zucker School of Medicine at Hofstra/Northwell, Department of Emergency Medicine, Hempstead, NY; 3 NYC Health + Hospitals, Department of Emergency Medicine, Brooklyn, NY; 4 Creighton School of Medicine Phoenix, Department of Emergency Medicine, Phoenix, AZ; 5 Mount Sinai Hospital, Department of Emergency Medicine, Brooklyn, NY; 6 Baylor College of Medicine, Department of Emergency Medicine, Houston, TX; 7 Boston University/Boston Medical Center, Department of Emergency Medicine, Boston, MA; 8 Montefiore Medical Center, Department of Emergency Medicine, Bronx, NY

## Abstract

**Audience:**

This case is meant for senior emergency medicine (EM) resident physicians as a preparatory tool for the American Board of Emergency Medicine Certifying Exam. However, it is applicable for EM residents at all levels of training.

**Introduction:**

Difficult conversations are an integral part of the practice of emergency medicine. Competent EM physicians routinely engage in challenging conversations with patients and their families (eg, delivering bad news, disclosing medical errors, providing death notifications).^1^ Despite their importance, communication skills are often not formally taught in residency training.^2^ Instead, learners frequently rely on role modeling to develop the skills necessary to navigate these difficult situations.^3^ Incorporating structured education and training around these challenging encounters has the potential to significantly enhance both learner preparedness and patient care.^2^,^4^,^5^

**Educational Objectives:**

This Observed Structured Clinical Examination (OSCE) is intended to cover the topic of Difficult Conversations. The overarching educational goal of this case is to assess learners’ communication skills, emotional responsiveness, and ability to facilitate a death notification conversation. Participants will be evaluated on their ability to communicate in an empathic, patient-centered manner while leading a difficult discussion. Successful participants will establish rapport, actively listen, disclose sensitive information clearly and compassionately, and respond appropriately to emotional reactions. By the end of the session, learners should be able to: (1) establish rapport with the patient’s family by initiating introductions and creating a supportive environment, (2) assess the family’s baseline understanding of the patient’s condition by using open-ended questions and active listening to elicit their perspective, (3) communicate the patient’s death clearly and compassionately, using concise, non-technical language, (4) demonstrate empathy by responding appropriately to emotional reactions, validating concerns, and addressing questions thoughtfully, and (5) provide closure to the conversation by summarizing key points, offering emotional support, and clarifying the next steps in the patient’s care.

**Educational Methods:**

We designed a single-station OSCE focused on the delivery of a death notification. This format is aligned with the Difficult Conversations module of the newly implemented ABEM Certifying Exam, which emphasizes communication skills and emotional responsiveness in a challenging simulated clinical encounter. In this OSCE, the learner is presented with a brief case summary describing the unsuccessful resuscitation of a patient in cardiac arrest and is tasked with delivering the bad news to the patient’s family. The family member is portrayed by a standardized participant who follows a structured script and provides standardized emotional and verbal cues. The examiner assesses learner performance using a detailed, behaviorally anchored checklist that includes both verbal and nonverbal communication skills. This OSCE structure mirrors the ABEM Certifying Exam to promote realism, consistency, and educational relevance.

**Research Methods:**

This simulation case was initially developed by three subject matter experts with backgrounds in EM and simulation-based education. To ensure clinical accuracy, coherence, and educational relevance, the case underwent a structured peer review process. Using the Simulation Scenario Evaluation Tool (SSET),^6^ a panel of three external reviewers evaluated the case and provided targeted feedback on elements such as case realism, scenario progression, clarity of learning objectives, and alignment with assessment metrics. Following peer review, the case was pilot-tested at two EM residency programs and a national EM academic conference. These pilot implementations aimed to evaluate the case’s clarity, feasibility, and instructional design within authentic educational environments. During these sessions, faculty facilitators and learners engaged with case materials, including a standardized participant script, examiner overview, and critical actions checklist. Feedback from this beta testing phase guided revisions to enhance standardized actor prompts, case logistics, and assessment materials.

**Results:**

Expert reviewers reported strong agreement that the learning objectives of the simulation case were specific, measurable, action-oriented, relevant, time-bound, and appropriately aligned with the experience level of the intended learners. They also noted that the clinical context, scenario progression, and integrated critical actions effectively supported these learning objectives. Faculty facilitators expressed strong agreement that the accompanying case materials and resources offered adequate guidance to support independent implementation of the case at their own institutions. Learner feedback indicated that both the written and verbal instructions were easy to follow and that the experience was valuable for preparing them for the ABEM Certifying Exam.

**Discussion:**

This simulation case effectively met its educational goals and proved to be a useful resource for preparing learners for the Difficult Conversations module of the ABEM Certifying Exam. Facilitators gave high ratings across key areas, emphasizing the clarity and quality of the learning objectives, scenario flow, and supporting materials. Learners consistently noted that the case offered valuable practice in communication skills and emotional engagement. These results support the use of structured OSCEs within EM residency curricula. Through simulation, this case contributes to closing educational gaps, fostering standardization, and improving learner readiness for board certification.

**Topics:**

Death notification, delivering bad news, communication, difficult conversations, American Board of Emergency Medicine, Certifying Exam.

**Table:** 

List of Resources:	
Abstract	14
User Guide	17
For Examiner Only	20
Certifying Exam Assessment	24
Stimulus	25
Debriefing and Evaluation Pearls	27

**Learner Audience:**

This case is appropriate for interns and junior and senior residents.

**Time Required for Implementation:**

Case: 10 minutes

Debriefing: 10 minutes

**Recommended number of learners per instructor:**

This case was specifically designed for one senior emergency medicine (EM) resident physician per instructor for individual practice to prepare for the American Board of Emergency Medicine (ABEM) Certifying Exam.

**Topics:**

Death notification, delivering bad news, communication, difficult conversations, American Board of Emergency Medicine, Certifying Exam.

**Objectives:**

By the end of the session, learners should be able to:

1. Establish rapport with the patient’s family by initiating introductions and creating a supportive environment
2. Assess the family’s baseline understanding of the patient’s condition by using open-ended questions and active listening to elicit their perspective.
3. Communicate the patient’s death clearly and compassionately, using concise, non-technical language
4. Demonstrate empathy by responding appropriately to emotional reactions, validating concerns, and addressing questions thoughtfully
5. Provide closure to the conversation by summarizing key points, offering emotional support, and clarifying the next steps in the patient’s care.

## Linked objectives, methods and results

The primary objective of this case is to familiarize EM residents with the expectations and format of the Difficult Conversations module of the ABEM Certifying Exam while allowing learners to practice and demonstrate their ability to facilitate a death notification. The Observed Structured Clinical Examination (OSCE) format was intentionally selected for its alignment with the ABEM Certifying Exam, providing a realistic environment to assess communication skills and emotional responsiveness. The case involves a 68-year-old male who presented in cardiac arrest and was pronounced dead after an unsuccessful resuscitation. The learner is expected to deliver a death notification to the patient’s wife. The case begins when the learner enters the room to meet with the patient’s wife. The learner should initiate introductions by clearly identifying themselves as the physician caring for the patient and confirming the family member’s relationship to the patient. They should create a supportive environment by offering the wife a seat and then sitting next to her (Objective 1). The learner should ask open-ended questions, employ active listening, and use non-verbal communication skills (eg, eye contact, head nodding, open body language) to assess the wife’s baseline understanding of the patient’s condition (Objective 2). The learner should concisely and compassionately review the patient’s clinical course and communicate the patient’s death, using clear, direct language (Objective 3). The learner should provide the wife with space and time for an emotional reaction and support her through the intentional use of silence and the offering of condolences and comfort (eg, tissues, water). The learner should validate the wife’s concerns and address her questions thoughtfully (Objective 4). Finally, the learner should provide closure to the conversation by summarizing key points, offering emotional support (eg, social worker, chaplain), and clarifying the next steps in the patient’s care (eg, viewing of the body, funeral services) (Objective 5).

## Recommended pre-reading for instructor

- Hobgood C, Harward D, Newton K, Davis W. The educational intervention "GRIEV_ING" improves the death notification skills of residents. *Acad Emerg Med*. 2005;12(4):296–301. doi: 10.1197/j.aem.2004.12.008
- Weaver L, Hobgood C. Death notification and advance directives. In: Tintinalli's Emergency Medicine: A Comprehensive Study Guide, 9th ed. McGraw-Hill; 2020:2017–2020.

## Results and tips for successful implementation

This case can be used either on its own or integrated into a full-length ABEM Certifying Exam practice session. To maximize realism, it is best suited for individual resident participation, though it can also be adapted for small group use across all training levels. Assigning learners to the facilitator role may enhance their understanding of the examiner’s approach. The case is intended to take approximately 10 minutes to complete, followed by a 10-minute debriefing for directed feedback.

To enhance learner engagement, we recommend conducting the case in a setting that closely resembles a typical ED room designated for private conversations with patients’ families. Whenever possible, a standardized participant should portray the patient’s family member, with the character’s identity adaptable to match the actor’s demographics. Alternatively, facilitators can assume the role of the family member, or the case can be delivered in an oral board format with learners verbalizing their clinical reasoning and planned actions.

The case was evaluated through an iterative pilot process involving a convenience sample of EM residents. Both faculty facilitators and resident participants completed anonymous surveys containing 5-point Likert scale questions (1 = strongly disagree, 5 = strongly agree) and open-ended questions. Survey responses were collected using Qualtrics (https://www.qualtrics.com) and analyzed using Excel (Microsoft, Redmond, WA). The Boston University Institutional Review Board reviewed the study and determined it to be exempt.

During the first round of testing, a facilitator at an EM residency program implemented the case with two EM residents. The facilitator used the Simulation Scenario Evaluation Tool (SSET)^6^ to assess the quality of the scenario, while residents completed a modified usability survey. A second round of testing took place at the Society for Academic Emergency Medicine Annual Meeting in May 2025 (Philadelphia, PA), and a third round was conducted at a different EM residency program. In these subsequent rounds, one facilitator and eight residents completed usability surveys. Revisions to the case were made following each round in response to survey feedback.

Feedback from the SSET was largely favorable. In the initial testing round, the facilitator strongly agreed that the case’s learning objectives were specific, measurable, action-oriented, relevant, time-bound, and appropriately aligned with the learners’ experience and knowledge base. The facilitator also strongly agreed that the clinical context, scenario flow, and critical actions were well-defined and effectively supported the intended learning objectives. In the second and third testing rounds, the facilitator (n = 1) rated the case highly across all evaluated domains, with mean scores of 4.0 out of 5 for ease of use and integration of material. They expressed interest in using the case for ongoing ABEM Certifying Exam preparation.

Resident evaluations (n = 9) mirrored these positive findings. Both written and verbal components of the case were consistently rated as clear, each with mean scores of 4.7. All respondents agreed or strongly agreed that the scenario was a valuable tool for exam preparation (mean = 4.9). Residents particularly noted the case’s realism and the clarity of its critical actions as key strengths.

## Figures and Tables

**Table:** Difficult Conversations Candidate Task Sheet

**CASE PARAMETERS**	
This is a 10-minute case. You will interact with a patient’s wife. You will deliver a death notification to the patient’s wife. Answer the wife’s questions and explain next steps.	
**PATIENT INFORMATION**	
**Patient Name**	Joe Johnson
**Age**	68-year-old
**Gender Identity**	male
**Presenting complaint**	Cardiac arrest
**General appearance/History of present illness**	Presented to ED in cardiac arrest, resuscitation unsuccessful
**Past Medical History**	Hypertension, hyperlipidemia, diabetes
**Medication**	Unknown
**Allergies**	Unknown
**Vital Signs on ED arrival**	Pulseless, apneic
**RESULTS/FINDINGS**	
Patient collapsed at home. Wife immediately performed chest compressions. On scene, initial rhythm was pulseless electrical activity (PEA). Prior to ED arrival, patient received 1mg epinephrine and a supraglottic airway was placed. In ED, initial rhythm was asystole. Patient underwent multiple rounds of CPR, received 3mg epinephrine, and was intubated. Point-of-care ultrasound showed no cardiac activity. Resuscitation terminated by you. Patient’s wife arrived at ED after patient already pronounced dead. She does not yet know that the patient has died.	
**TASK STATEMENT**	
Your task is to deliver a death notification to the patient’s wife.	

## FOR EXAMINER ONLY

### Difficult Conversations Case: Death Notification Summary

**Diagnosis:** Cardiac arrest

**Case Summary:** A 68-year-old male presented to the ED in cardiac arrest and was pronounced dead after an unsuccessful resuscitation. The learner must deliver a death notification to the patient’s wife.

**Standardized Actor Profile:**

The standardized actor will portray the patient’s wife, who is a 65-year-old female who has been married to the patient for 40 years. They have a 35-year-old daughter who lives in a different state.

**Materials/personnel needed:**

The case will require one standardized participant, two chairs, and a box of tissues. If a standardized participant is not available, an instructor can play the patient’s spouse instead.

**Room Setup:**

The space should be set up as a standard ED room that would typically be used for private conversations with patients’ families.

**Examiner Summary:**

The patient was a 68-year-old male with a history of hypertension, hyperlipidemia, and diabetes who presented to the emergency department by emergency medical services (EMS) in cardiac arrest.

The patient had been experiencing intermittent chest pain and shortness of breath for the past week but had not sought a medical evaluation for his symptoms. Today, he did not feel well enough to go on his usual morning run, so he decided to mow the lawn instead. When he finished and came back inside, his wife noticed that he was pale and diaphoretic. While walking across the living room, he collapsed to the floor. His wife heard a thud and responded immediately. She noticed that he was not breathing and did not have a pulse, so she called 911 and initiated chest compressions.

Upon EMS arrival, the patient’s wife was performing chest compressions. The patient was pulseless, apneic, but still warm to touch. His initial rhythm showed PEA. Intraosseous access was established, and the patient received 1mg of epinephrine. A supraglottic airway was placed. The patient was placed in the ambulance with ongoing chest compressions and transported to the emergency department (ED).

In the ED, the patient’s initial rhythm showed asystole. He underwent multiple 2-minute rounds of chest compressions and received 3 additional doses of 1mg epinephrine. Endotracheal intubation was performed, and the patient was mechanically ventilated without difficulty. Point-of-care glucose was normal. The patient remained in asystole throughout the resuscitation. The resuscitation was terminated by the emergency physician (learner).

The patient’s wife did not accompany the patient in the ambulance and drove to the ED separately. She has just arrived at the ED, after the patient has already been pronounced dead. She does not yet know that her husband has died.

**Play of Case**

The case starts with the learner entering the room. The learner should initiate introductions and establish rapport. They should ask open-ended questions and employ active listening to assess the wife’s baseline understanding of the patient’s condition. They should clearly and compassionately review the patient’s ED course and communicate that the patient has died. They should provide the wife with space and time for an emotional reaction, supporting them and offering condolences. They should answer the wife’s questions and clarify next steps.

### Difficult Conversations Case: Death Notification Standardized Patient Case Summary and Script

Your husband is a 68-year-old man with high blood pressure, high cholesterol, and diabetes. For the past week, he has been experiencing intermittent chest pain and shortness of breath. He has not seen a healthcare practitioner for these symptoms. Today, he did not feel well enough to go on his usual morning run, so he decided to mow the lawn instead. When he finished and came back inside, you noticed that he looked pale and sweaty. A few minutes later, you heard a thud in the living room. You immediately went to check and found your husband lying on the floor. He was unresponsive, did not appear to be breathing, and did not seem to have a pulse. You started chest compressions and called 911.

When the paramedics arrived, they took over chest compressions. You watched them put pads on his chest, and you heard them say that he had a “nonshockable rhythm.” They drilled something into his shoulder and gave him medicines. They put a tube into his mouth to help him breathe. They put your husband in the ambulance and told you which hospital he would be transported to. You did not immediately leave your house because you wanted to first call your daughter (a pediatrician) to let her know what had happened. Then you got into your car and drove to the hospital.

When you arrived at the emergency department (ED), you were greeted by a social worker, who brought you to the family room and told you that the emergency physician (learner) would be coming in a few minutes to give you an update.

**Play of Case:**

While waiting for the learner, you are initially seated. As the learner enters the room, you will stand up and immediately begin to inquire about your husband: “How is my husband? Is he okay? Please tell me he’s okay.”

The learner should introduce themselves as “Doctor” and confirm your relationship with the patient. They should encourage you to sit down. If they do not ask you to sit down, you will continue to pace around the room, becoming increasingly anxious. Eventually, you will sit down when the learner informs you that your husband is dead.

The learner should ask you an open-ended question about what you currently know about what is going on with your husband. You will provide a brief description of his recent symptoms, his appearance today, and what happened after he collapsed in the living room.

The learner may then describe the events in the ED. They should use plain language to describe the ED course and the interventions that were performed. If the learner uses any medical jargon or abbreviations, you will ask for clarification.

Then, the learner should clearly and compassionately communicate that your husband has died. If the learner does not use the word “died” or “dead,” you will not understand what the learner is trying to convey and will continue to repeat: “So is he okay? Please tell me he’s okay.”

After hearing this information, you will break down and cry. The learner should give you space and time to have an emotional reaction. They may offer to provide you with tissues or offer condolences.

After this emotional reaction, you will begin to ask questions and make statements:

- “How could this have happened? Why did he die?”
- “What could I have done differently?”
- “I should have tried harder to get him to see a doctor when he started having chest pain a week ago. He is stubborn so I didn’t push it. It’s all my fault.”
- “Did he suffer?”

Finally, the learner should bring the conversation to an end by clarifying next steps. If they have not made this transition by the last minute of the case, you will ask/state:

- “What am I going to do now?”
- “Can I see my husband?”
- “I need to call my daughter.”

## CERTIFYING EXAM ASSESSMENT

*Difficult Conversation Case: Death Notification*

Learner: _________________________________________

### Establish rapport with patient’s family member

□ Introduce self as “Doctor”
□ Identify patient and confirm family member’s relationship to patient
□ Ask family member to sit down and then sit down beside family member

### Assess family member’s baseline understanding of patient’s condition

□ Ask open-ended questions (eg, “What do you know about what is going on with your loved one?”)
□ Demonstrate active listening using non-verbal communication techniques (eg, eye contact, head nodding, open body language)

### Communicate patient’s death clearly

□ Be concise
□ Use plain language
□ State “he died,” or “he has died,” or “he is dead”

### Demonstrate empathy

□ Support family member (eg, remain seated next to family member, provide tissues/water)
□ Offer condolences and respond appropriately to emotional reactions
□ Validate any concerns they have

### Provide closure to conversation

□ Answer family member’s questions
□ Clarify next steps in patient’s care
□ Offer emotional support (eg, social worker, chaplain)

**Summative and formative comments:**

### Stimulus Inventory

**Candidate Task Sheet**

## DEBRIEFING AND EVALUATION PEARLS

### Death Notification

Delivering bad news is a commonly performed task that many clinicians find challenging. Common pitfalls include the use of medical jargon, divided attention due to competing demands in the emergency department, and poor nonverbal communication (eg, standing rather than sitting, failure to find a quiet, calm environment for the conversation).^7^,^8^ Several structured mnemonics are available to help guide learners through the process, and these frameworks should serve as a foundation for the debriefing.

#### SPIKES^9^

S - Setting

- Choose a private, comfortable, nonthreatening setting
- Invite important people
- Minimize distractions (eg, silence phones)
- Sit down and make eye contact

P - Perception

- Uncover what the patient/family thinks is happening
- “Tell me what you understand about your medical situation”

I - Invitation

- Ask patient/family what they would like to know

K - Knowledge

- Warn patient/family that they are about to receive some bad news
- Explain disease and care options in plain language

E - Emotion and Empathy

- Respect feelings and respond with empathy

S - Strategy and Summary

- Recap information and discuss next steps
- “We’ve talked about a lot of things. Can you please tell me what you understand?”

#### GRIEV_ING **(**specific to death notification)^10^

G - Gather

- Gather family, ensure all important members are present

R - Resources

- Call for support services to assist family (eg, social worker, chaplain, alternate family/friends)

I - Identify

- Identify yourself and your role
- Identify the patient by name
- Identify the family’s state of knowledge

E - Educate

- Explain to the family the events that have occurred to the patient and tell them about the patient’s current state

V - Verify

- Verify the patient has died
- Use the word “dead” or “died”

_ - Space

- Give space for family to have an emotional response and absorb the news

I - Inquire

- Ask if there are any questions; answer as many of them as you can

N: Nuts and bolts

- Discuss funeral services
- Inquire about personal belongings
- Offer family the opportunity to view the body

G: Give

- Give contact information for resources to help family through the grieving and death process

**The debriefing on this case should focus on:**

- Verbal communication:
  - Use of open-ended versus closed-ended questions
  - Use of plain language versus medical jargon
  - Use of precise language (eg, “dead” or “died”)
  - Use of active listening and phrase of acknowledgement
- Nonverbal communication:
  - Did learners use body language to portray openness and connection to the situation (eg, sitting at the level of the family, leaning forward, avoiding crossed arms)?
  - Did learners employ silence strategically to allow the family to have an emotional response?
  - If the standardized participant is present for debriefing, include time to allow them to speak on how they felt as the family member receiving the news and whether they felt heard and supported by the learner

#### Debriefing Plan - PEARLS Framework

- Reactions Phase
  *Goal: Allow the learner to process initial emotions.*
  - "How did you feel about the case?"
  - "What went well? What did not go well?"
- Description Phase
  *Goal: Establish a shared understanding of the case.*
  - "In 2–3 sentences, please summarize this case."
- Analysis Phase
  *Goal: Facilitate reflection and provide structured feedback based on rubric scores.*
  - "What was your strategy for structuring the death notification discussion?"
  - "How did you establish rapport with the patient’s family member?"
  - "How did you respond to the family member’s emotional reaction?"
  - "Did the conversation go smoothly? What would you do differently if you encountered this case again?"
- Application/Summary Phase
  - "What is one key thing you learned today that you will apply the next time you perform a death notification?"
